# The Late Baron Larrey

**Published:** 1842-10-01

**Authors:** 


### THE LATE BARON LARREY.

The death of Baron Larrey, which took place at Lyons on the 29th of July, has deprived French Surgery of one of its brightest ornaments. Few members of our profession have played so important and distinguished a part in the annals of both history and science, as the veteran Larrey. His career was connected with some of the most stirring events of the past half century, and his reputation belongs hardly more to his profession than to humanity.

Jean-Dominique Larrey was born in 1776, in the village of Baudeau, in France. Left early an orphan, at the age of fifteen he was placed under the care of a paternal uncle, who practised surgery at Toulouse, with whom he spent seven years in the study of medicine. In 1787, he obtained the post of Surgeon in the Navy, and made a cruise to the colonies. Returning shortly afterwards to France, in the midst of the excitement of the revolution, he became attached, as an *Interne*, to the hospital of the Invalids. After remaining here some years, he obtained the rank of full-surgeon, and joined the army of the Rhine. Larrey soon signalized himself in the army by the introduction of the *ambulances volantes*, the improved transports for hospital stores, which have been found so useful in the French service. Larrey's services and his *ambulances volantes* were considered of so much importance, that, after the battle of Mayence, in 1793, they were publicly noticed in the bulletin, issued by General Beauharnais.

In 1794, Larrey was appointed Surgeon-General, and repaired to Toulon, where commenced his intimacy with Napoleon, then a lieutenant of artillery. The school of military surgery at the Val-de-Grâce had been previously established, and Larrey appointed a Professor; but professors and pupils were soon called to active service, and the school was suspended. In 1798, Larrey joined the army of Egypt. His account of the memorable campaign in Egypt and in Syria is familiar to every professional reader. Larrey accompanied Napoleon in nearly all his campaigns, and was honored by him with repeated marks of distinction. In 1812, he received the title of Surgeon-General of the *Grande Armée*. His fidelity to the emperor brought him into active service, during the hundred days, and he was present at Waterloo, where he was wounded and made prisoner. During the restoration, M. Larrey's well known attachment to Napoleon, kept him under a cloud, but the revolution of 1830 restored him to the public service, to which he devoted himself to the close of his life. His death at Lyons took place upon his journey home from the army in Algeria.

Baron Larrey's scientific was not less distinguished than his military career. His writings on military surgery have been long recognized as the highest authority on the subject, and certainly no surgeon of his day equalled him in the amount and variety of his contributions to practical surgery. In 1803 he published his "*Historical and Surgical Account of the Campaign of the Army of the East, in Egypt and Syria*." In 1812, he published three volumes of his admirable "*Memoirs of Military Surgery and Campaigns*," adding a fourth volume in 1817. Besides these, Larrey published numerous other works, including four volumes of "*Surgical Clinique derived chiefly from Camps and Hospitals, from* 1792 *to* 1832."

The merit of the adaptation of the *immoveable apparatus* to the treatment of fractures and certain wounds of the soft parts, belongs, we believe, to M. Larrey. The advantages of this mode of treatment in the surgery of actual warfare can hardly be disputed, whatever may be our views of the practice under ordinary circumstances. In his last work, published shortly before his death, M. Larrey gives a new notice of the apparatus, and sets in a striking point of view, the advantages of its adoption in military surgery.

Baron Larrey's writings possess the double attraction of historical and scientific value. An actor in scenes, the grandeur of which has never been surpassed, his narrations constitute not only invaluable records of practical surgery, but they, at the same time, form an interesting chronicle of a most eventful epoch of European history. On the great merit of Larrey's writings on military surgery, it is unnecessary to dwell. His immense clinical experience, derived from a theatre that has never been equalled in magnitude, gives them an importance and authority that it would be presumption to question. He was perhaps, in some respects, too pertinacious an adherent of the opinions of by-gone times, and did injustice to the importance of recent discoveries, but his works are all entitled to the praise of practical value, and are distinguished by a lucid detail of facts, a just discrimination of their relative importance, and the evidences of a sound judgment.

Larrey's career was as virtuous as it was memorable. He enjoyed, pre-eminently the confidence and respect of Napoleon, who spoke of him at St. Helena in the warmest terms. His allusion to him in his will is well known.

Baron Larrey was interred at Père-la-Chaise, with great solemnity. A number of distinguished men followed him to the tomb, and orations were delivered by MM. Pariset, Breschet, and others. A subscription has been opened to erect a monument to his memory.

